# Sampling a trillion-sized GDB-20 database of drug-like molecules by generative artificial intelligence

**DOI:** 10.1039/d6dd00213g

**Published:** 2026-10-16

**Authors:** Ye Buehler, Sacha Javor, Jean-Louis Reymond

**Affiliations:** a Department of Chemistry, Biochemistry and Pharmaceutical Sciences, University of Bern Bern 3012 Switzerland jean-louis.reymond@unibe.ch

## Abstract

Identifying new chemical entities for small molecule drug discovery requires the continued exploration of its vast chemical space, which has been speculated to contain 10^60^ or more molecules. Here we used generative artificial intelligence to sample a GDB-20 database estimated to contain 32 trillion organic molecules corresponding to 26.9 million molecular graphs up to 20 nodes selected for their limited topological complexity. We show that GDB-20 populates a distinct chemical space of sp^3^-rich, 3D-shaped and natural product-like molecules with favorable synthetic accessibility score. The relevance for drug discovery of our 12 billion sample, named GDB-20s and corresponding in size to approximately 0.04% of the estimated full database, is suggested by high compliance with drug-likeness and toxicity filters, and the coverage of thousands of scaffolds occurring in bioactive molecules recorded in the ChEMBL database. The generative models used to produce GDB-20s, together with the 12-billion-molecule collection are available for download at https://gdb.unibe.ch.

## Abstract 2

Generative AI sampled 12 billion GDB-20 molecules, revealing an sp^3^-rich, 3D-shaped and natural product-like chemical space with potential synthetic accessibility, drug-likeness, toxicity-filter compliance and broad bioactive scaffold coverage.

## Graphical abstract

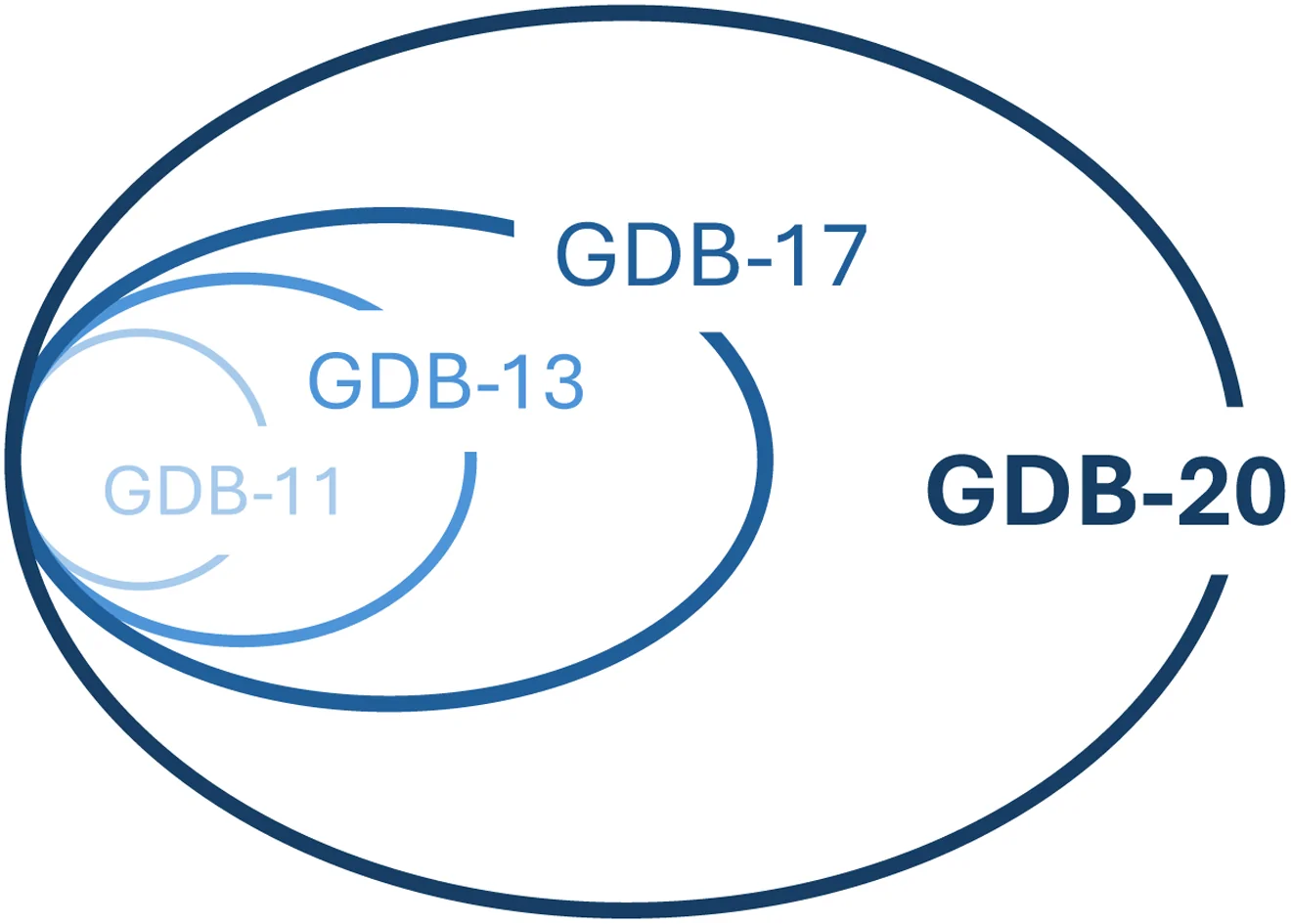

## Introduction

Since the advent of combinatorial chemistry and related approaches, early-phase drug discovery has relied on increasingly large collections of molecules for both experimental and virtual screening.^1–5^ Although these collections reach into the trillions, they only represent a very small fraction of the drug-like chemical space estimated to contain up to 10^60^ molecules,^6–8^ simply because molecules in these collections result from the combinatorial assembly from at most a few hundred thousand building blocks,^9–12^ which themselves only represent an almost insignificant fraction of the theoretical possibilities. For instance, we have previously used systematic enumeration from first principles taking into account ring strain and functional group rules to focus on feasible molecules,^13,14^ and obtained the generated databases GDB-11 (26.4 million molecules up to 11 atoms of C, N, O, F),^15,16^ GDB-13 (970 million molecules up to 13 atoms of C, N, O, S and Cl)^17,18^ and GDB-17 (166 billion molecules up to 17 atoms of C, N, O, S and halogens).^19,20^ These GDBs outnumber by orders of magnitude known molecules of similar size such as those collected in open access databases such as ChEMBL (bioactive molecules),^21^ COCONUT (natural products),^22^ ZINC (commercial screening compounds),^23^ or PubChem (aggregated data of public datasets),^24^ even when restricted to fragment-like^25,26^ and drug-like subsets,^27,28^ ring systems,^29^ molecular frameworks^30^ or ring fragments.^31^

The GDBs populate a chemical space of chiral, 3D-shaped molecules which might help overcome limitations of aromatic and heteroaromatic ring systems^32,33^ in terms of solubility, metabolism, polypharmacology and toxicity.^34,35^ Besides providing direct evidence for the size and nature of the small molecule chemical space, the GDBs are interesting for drug discovery because innovative molecules can be directly identified by virtual screening or by novelty selection from dedicated GDB subsets for synthesis and testing.^36–45^ To further support this effort, we report the extension of our GDB approach to chemical space up to a molecular size of 20 non-hydrogen atoms (heavy atom count, HAC ≤ 20), further enlarging the space covered by our previous databases by several orders of magnitude.

To limit database size, which was expected to increase by at least 10-fold for each additional non-hydrogen atom in an unconstrained model, we strongly restricted molecular complexity at the level of the molecular graphs, the types of functional groups, and the overall molecular polarity taking into account the structural types encountered in drug molecules.^46^ Even under such constraints, our GDB-20 was estimated to contain 32 trillion molecules. To explore its contents without going through a resource intensive and impractical full enumeration, we set out to use generative artificial intelligence. Starting with a selection of mathematical graphs up to 20 nodes produced by the program GENG,^47^ we generated an initial set of GDB-20 molecules using a graph-to-molecule transformer model trained with molecules from our previous GDBs. We then used various subsets of these GDB-20 molecules to train recurrent neural networks (RNN) with Long Short-Term Memory (LSTM) recurrent cells, from which we sampled additional GDB-20 molecules. Such RNNs were previously shown to efficiently sample chemical space at the example of GDB-13,^48,49^ and have inspired the generative component of the REINVENT framework.^50^ The combined output of the graph-to-molecule transformer and RNN generative models produced an overall sample of 12 billion molecules (corresponding in size to approximately 0.04% of the full database), here called GDB-20s (Fig. 1). The relevance of GDB-20s for drug discovery is indicated by high compliance with drug-likeness and toxicity filters, good synthetic accessibility scores, a high degree of novelty, and the coverage of thousands of scaffolds occurring in bioactive molecules recorded in the ChEMBL database.

**Fig. 1:**
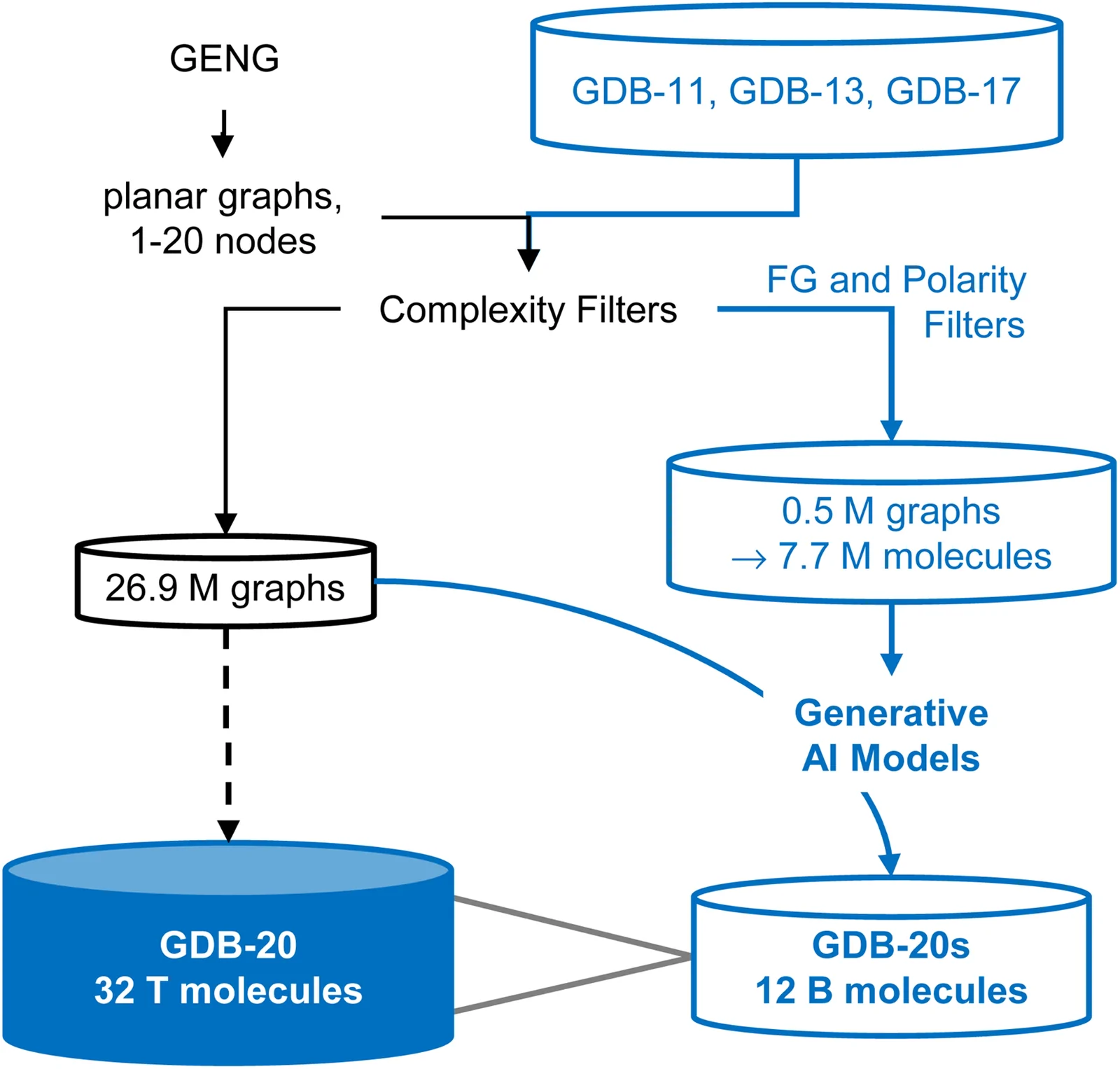
Graph selection procedure and workflow for generating the GDB-20s sample of GDB-20. Graph complexity filters: ≤3 rings, ring size 5–8, no node shared by three rings, at least 40% divalent nodes (MC1 < 0.6). FG filters: remove molecules containing non-aromatic C=C, C=N, N–N, N–O, and O–C–O. Polarity filter: *c* log *P* > 0. Generative artificial intelligence (AI) models comprise a transformer to obtain GDB-20 molecules from graphs, and recurrent neural networks (RNNs) to sample additional GDB-20 molecules.

## Results and discussion

### Graph selection and database size estimation

We started our enumeration with the 5.9 billion topologically planar graphs (graphs that can be drawn in 2D without crossing edges) up to 20 nodes of maximum node valency four without any 3- and 4-membered rings, as produced the program GENG.^47^ From this list, we selected graphs with up to three rings containing at most one seven- or eight-membered ring and no larger ring, and in which no node belongs simultaneously to three rings. We further restricted complexity by requesting at least 40% divalent nodes (MC1 < 0.6),^46^ which excluded highly branched graphs containing multiple trivalent and tetravalent nodes. This procedure left a selection of 26.9 million graphs deemed to mostly correspond to synthetically tractable structures. Extrapolating from the number of molecules per graph in our previous database GDB-17,^19^ we estimated that the GDB-20 dataset resulting from enumerating molecules from these 26.9 million graphs should contain approximately 32 trillion molecules (Table 1 and Fig. S1).

**Table 1:** Graph generation, filtering statistics, and estimated chemical space size of GDB-20

Nodes^a^	GENG output^b^	Graphs passing ring filters^c^	Graphs passing complexity filter^d^	Mols/graph^e^ in GDB-17	Size estimate GDB-20^f^
1–13	36 237	14 942	7003	1163	8.1 M
14	100 517	34 779	17 178	3883	67 M
15	768 629	118 902	42 192	15706	660 M
16	4 309 923	410 753	172 563	28556	4.9 B
17	24 870 439	1 422 300	681 416	69127	47 B
18	146 455 927	4 929 744	1 737 129	217 K*	380 B
19	875 349 668	17 024 485	6 844 189	588 K*	4.0 T
20	4 882 045 873	58 516 294	17 366 489	1.59 M*	28 T
SUM	5 933 937 213	82 472 199	26 868 159	—	32 T

a Number of nodes considered.

b Number of planar graphs generated by GENG, excluding 3- or 4-membered rings.

c Ring filters: at most three rings; no atom shared by three rings, maximum one 7- or 8-membered ring and no ring larger than 8-membered.

d Molecular complexity MC1 < 0.6. MC1 = 1 − FDV; FDV is the fraction of divalent nodes in the molecular graph.

e Number of molecules per graph in GDB-17; values for nodes 18–20 from exponential extrapolation, see Fig. S1 (marked with *).

f Estimated number of molecules in GDB-20.

### Graph-to-molecule transformer

To generate GDB-20 molecules from the graphs, we trained a transformer model to translate the saturated hydrocarbon equivalent of these graphs, written in SMILES format,^51^ into the corresponding molecules, a type of transformation for which transformer models are well suited.^52–54^ To train our transformer, we selected molecules from our previously reported GDB-11 (26.4 M), GDB-13s (99 M), and a random subset of GDB-17 (50 M) fitting the above graph criteria as well as polarity (*c* log *P* > 0) and functional group criteria (non-aromatic C=C, C=N, N–N, N–O and O–C–O were removed). Keeping a maximum of 300 molecules per graph and selecting molecules to reach an overall element composition resembling that of ChEMBL produced a training set of 7 653 204 molecules derived from 511 304 graphs.

For each translation of a hydrocarbon graph into a molecule in the above dataset, we used the canonical SMILES notation of the molecule and generated the corresponding saturated hydrocarbon SMILES by eliminating bond types and changing all atoms to non-aromatic carbon. An initial transformer model trained with this dataset to produce diverse molecules from saturated hydrocarbons, however, returned only approximately 92% valid SMILES when challenged with GDB-20 saturated hydrocarbons with 18–20 atoms. We tentatively attributed this problem to the fact that SMILES for these larger hydrocarbons, which composed 94% of the GDB-20 set, were significantly longer than those in the training dataset and therefore beyond the applicability domain of the model. We therefore grouped two thirds of the hydrocarbons in the training set as concatenated SMILES pairs, each pair being transformed to the corresponding concatenated SMILES pair of molecules. The SMILES length distribution of the hydrocarbons in this modified training set covered the length range of our GDB-20 hydrocarbons much better (Fig. S2).

Indeed, a transformer model trained with this modified training set produced 97.4% valid SMILES when challenged with GDB-20 hydrocarbon SMILES and was used to sample 300 molecules per graph for each of the 26 868 159 selected graphs. SMILES canonicalization and removal of duplicates reduced the output to 67% of the valid SMILES produced by the transformer model (Tables 2 and S1). Almost all molecules produced (98.4%) satisfied the GDB-20 selection criteria applied to the training set, and 99.99% of a random sample of one million transformer outputs followed the functional group selection rules. Note that 873 427 (11.4%) of the 7 653 204 molecules up to 17 atoms used in the transformer train/validation set were regenerated by the sampling procedure. Taken together, the transformer sampling procedure above produced 5 292 803 769 molecules (0.017%) of the expected 32 trillion dataset of GDB-20.

**Table 2:** Performance of the hydrocarbon-to-molecule transformer model

HAC^a^	1–17	18	19	20	Overall
SMILES Gen^b^	276 105 600	521 138 700	2 053 256 700	5 220 891 300	8 071 392 300
Valid^c^	268 398 911	508 670 499	2 000 420 938	5 081 151 301	7 858 641 649
% Valid	97.21%	97.61%	97.43%	97.32%	97.36%
Unique^d^	213 339 460	374 215 329	1 413 619 876	3 291 629 104	5 292 803 769
% Unique	79.49%	73.57%	70.67%	64.78%	67.35%

a Heavy-atom count in the hydrocarbon SMILES corresponding to the graphs.

b Number of SMILES generated from transformer model. Only 4930 molecules correspond to one of 678 graphs not present in the original collection of 26.9 million graphs, either due to a small ring or to a low fraction of divalent nodes (MC1 filter).

c Number of SMILES successfully parsed by RDKit.^55^

d Number of unique molecules among all valid generations. No tautomer standardization is applied; distinct tautomeric forms are treated as unique entries to preserve the raw generative output. Molecules violating the ‘ideality’ rules are retained in the released set because they remain chemically sound and may be valuable for specific use cases. In a 1 million random sample, a few molecules broke the FG rules: 51 molecules featured an –O–C–O– group, 121 molecules contained non-aromatic double bonds, 7 molecules contained a non-aromatic NN or NO bond, and one molecule contained a non-aromatic sulfur atom. Performance values of the transformer are presented in Table S1.

### RNN sampling of additional GDB-20 molecules

The initial GDB-20 dataset above obtained by the graph-to-molecule transformer could now be used to obtain additional GDB-20 molecules by training a Recurrent Neural Network (RNN) architecture with Long Short-Term Memory (LSTM) recurrent cells using the procedure previously reported for GDB-13.^48,49^ Taking into account the very large dataset size anticipated for GDB-20, we trained five different RNN models covering the molecule size range and sampled each model for a total of 10 billion generated SMILES, which after removal of invalid SMILES, canonicalization and removal of duplicates, left 8.1 billion unique molecules (Tables 3 and S1).

**Table 3:** Intrinsic evaluation of generated SMILES across graph node subsets for the RNN generative models

Nodes^a^	1–17	18	19	20	Overall
SMILES Gen^b^	1 000 000 000	2 000 000 000	3 000 000 000	4 000 000 000	10 000 000 000
Valid^c^	997 240 946	1 992 913 306	2 989 646 973	3 983 657 434	9 963 458 659
% Valid	99.72%	99.65%	99.65%	99.59%	99.63%
Unique^d^	625 369 415	1 351 372 854	2 548 986 442	3 621 520 000	8 147 248 711
% Unique	62.71%	67.81%	85.26%	90.91%	81.77%

a Each RNN was trained using one million molecules in training set and one million molecules in validation set, selected randomly from the corresponding group and written as randomized SMILES. Number of graph nodes considered.

b Number of SMILES generated from RNN generative models.

c Number of SMILES successfully parsed by RDKit.^55^

d Number of unique molecules among all valid generations. No tautomer standardization is applied; distinct tautomeric forms are treated as unique entries to preserve the raw generative output. Molecules violating the ‘ideality’ rules are retained in the released set because they remain chemically sound and may be valuable for specific use cases. In a 1 million random sample of the RNN outputs, 508 molecules featured an –O–C–O– group, 203 molecules contained non-aromatic double bonds, 43 molecules contained a non-aromatic NN or NO bond, and 4 molecules contained a non-aromatic sulfur atom. Performance values of the RNN models are presented in Table S1.

Although trained on only one million molecules each, the combined output of the five RNNs contained molecules corresponding to 31 498 589 hydrocarbon graphs. These graphs covered 99.8% of the 26.8 million graphs selected for GDB-20. The 14.9% additional graphs, accounting for 5.7% of all RNN generated molecules, broke the GDB-20 graph selection rules either because they featured a small ring, or a low fraction of divalent node (MC1 filter), or because they contained a larger than 8-membered ring. On the other hand, 99.95% of a random sample of one million RNN outputs followed the functional group selection rules. Together with the transformer-generated molecules that violated the GDB-20 rules, these molecules were retained because the violations do not imply chemical invalidity and because the structures may be useful for exploring chemical space immediately beyond the GDB-20 boundaries; however, they should not be considered strict members of the rule-defined GDB-20 space (noncompliant GDB-20s records can be identified using the undesired_FG_check() function provided in the released code, which applies the functional-group, structural, and polarity criteria).

### Novelty, diversity and molecular properties of GDB-20s

The combination of the graph-to-molecule and RNN models discussed above resulted in a collection of 12 092 137 338 unique molecules (following restriction to the target HAC range, the transformer and RNN outputs contained 5 292 756 714 and 8 133 312 955 unique molecules, respectively, of which 1 333 932 331 occurred in both outputs and were counted only once), which was considered for further characterization and referred to as GDB-20s. Although representing only 0.04% of the estimated 32 trillion molecules of the full database, this dataset exceeded the number of known molecules of similar molecular size in public databases such as ChEMBL (bioactive molecules),^21^ COCONUT (natural products)^22^ and ZINC (commercial compounds),^23^ by several orders of magnitude and therefore contained almost entirely novel molecules (Table 4, upper part). Estimation of dataset diversity using iSIM (instant similarity)^56^ gave higher iSIM values for GDB-20s compared to reference datasets, indicating that GDB-20s molecules were on average more similar to each other and therefore a denser coverage of chemical space. Furthermore, the iSIM-σ values were higher for GDB-20s than for reference datasets, indicating a broader coverage of chemical space.^57^

**Table 4:** Comparison of GDB-20s with ChEMBL, COCONUT and ZINC by number of molecules and scaffolds up to 20 atoms

	GDB-20s^a^	ChEMBL^b^	COCONUT^c^	ZINC^d^
Unique molecules	12 092 137 338	2 409 270	63 971	883 893 331
Unique molecules ≤20	12 092 137 338	355 421	20 968	78 926 952
iSIM^e^	0.2725	0.1794	0.1712	0.1863
iSIM-σ^e^	0.1086	0.0505	0.0938	0.0613
Unique scaffolds	72 101 443	712 179	21 375	41 424 676
Unique Scaffolds ≤20	72 101 443	176 840	9 806	4 366 195
Shannon entropy of scaffold	13.6773	12.1190	12.7292	12.0489
iSIM^e^	0.2229	0.1744	0.1810	0.1859
iSIM-σ^e^	0.0785	0.0529	0.0937	0.0502

a Generated sample representing 0.04% of the estimated full database.

b ChEMBL version 34.

c Only considering natural products annotated with a doi: number.

d Commercial screening compounds.

e Calculated using RDKit binary fingerprints with a bit length of 2048.

A TMAP visualization^58^ of random samples of molecules up to 20 non-hydrogen atoms from GDB-20s (14 911), ChEMBL (15 017), COCONUT (6 222) and ZINC (15 147) organized by substructure similarity using the MAP4C fingerprint^59^ showed that GDB molecules occupied a distinct region of chemical space (Fig. 2a). Property profiling of 50 000 molecules up to 20 non-hydrogen atoms from each dataset showed the expected steeper increase in the number of molecules as function of size compared to reference datasets (Fig. 2b and S3). On the other hand, only GDB-20s contained a significant number of 7- and 8-membered ring compounds and a higher fraction of tricyclic molecules compared to the three reference datasets (Fig. 2c and d). GDB-20s had a similar rotatable bond count profile to ChEMBL ≤ 20 (Fig. 2e), but compared to all three reference datasets, a much higher number of stereocenters (Fig. 2f) and fraction of sp^3^ centers (*F*_sp^3^_, Fig. 2g), resulting in a higher natural product-likeness score (NPscore, Fig. 2h).^60^ Along with high *F*_sp^3^_ values, GDB-20s molecules were generally more 3D-shaped than those in reference databases as measured by the principal moment of inertia of 3D-models (Fig. S4).^61,62^ The synthetic accessibility scores (SAscore) of GDB-20s molecules were significantly higher than for reference datasets, indicating that the molecules were composed of substructures mostly not found in known molecules and therefore assessing their high degree of novelty. Nevertheless, GDB-20s SAscore values were mostly below 6, indicating potential synthetic accessibility (Fig. 2i).^63^

**Fig. 2:**
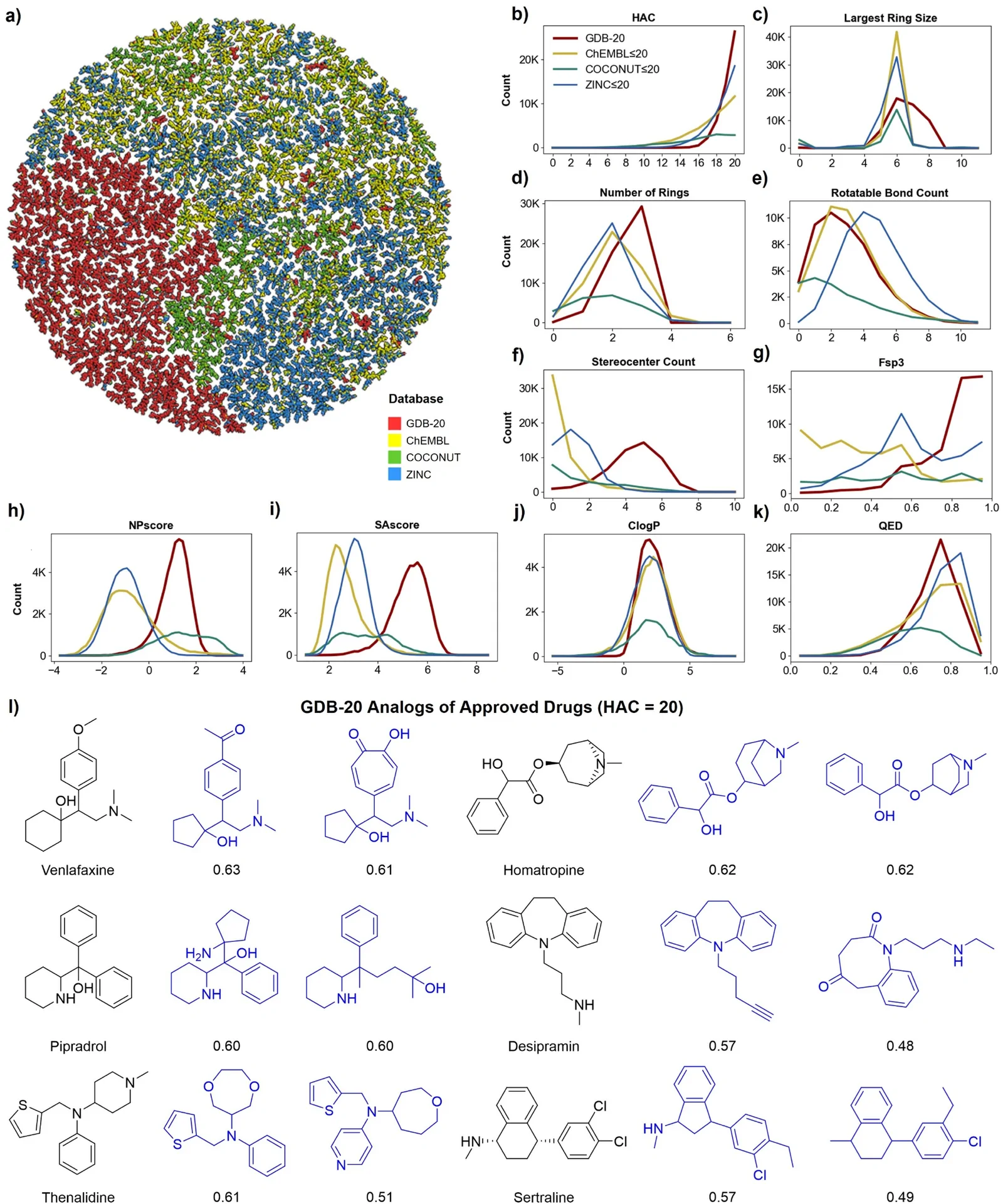
Diversity and properties of GDB-20s molecules (a) TMAP visualization of a combination of random samples of molecules up to 20 non-hydrogen atoms from GDB-20s (14 911), ChEMBL (15 017), COCONUT (6 222) and ZINC (15 147), color-coded by dataset source, organized by substructure similarity using the MAP4C fingerprint. An interactive version of the TMAPs with additional color-codes is accessible at https://tm.gdb.tools/MAP4C/GDB-20_50K_molecules. (b–k) Distribution of key molecular properties in GDB-20s compared with reference compound collections capped at HAC ≤ 20. Each panel shows the property distributions for GDB-20s (red), ChEMBL ≤ 20 (yellow), COCONUT ≤ 20 (green), and ZINC ≤ 20 (blue), using 50 000 randomly selected molecules from each dataset (COCONUT ≤ 20: all 20 968 eligible molecules). Properties include heavy atom count (HAC), largest ring size, number of rings, rotatable bonds and stereocenters, fraction of sp^3^ carbons (*F*_sp^3^_), natural product-likeness score (NPscore), synthetic accessibility score (SAscore), fragment-based calculated log *P* (*c* log *P*), and quantitative estimate of drug-likeness (QED). (l) Analogs of selected approved drugs (HAC = 20) with the highest Tanimoto similarity to their corresponding DrugBank parent molecules, calculated using ECFP4 fingerprints (radius = 2, nBits = 2048). The parent drugs are shown on the left, and their most similar analogs identified in GDB-20s are displayed to the right. The Tanimoto similarity values are indicated below each analog structure. None of the analogs listed are known (Reaxys search).

The number and diversity of molecules available in GDB-20s was well illustrated by the presence of a very large number of constitutional isomers of known drugs. For example, from 73 approved drug featured in DrugBank with HAC = 20, we identified between 29 (chloramphenicol, C_11_H_12_Cl_2_N_2_O_5_) and 181 093 992 (venlafaxine, C_17_H_27_NO_2_) isomers in our GDB-20s dataset (Fig. S5a and Table S2). As exemplified in a TMAP of a random 50 000 selection of venlafaxine isomers, these constitutional isomers were very diverse (Fig. S5b). On the other hand, virtual screening of our 12 billion GDB-20s molecules for high Tanimoto similarity analogs of known drugs returned only very few hits, reflecting the very broad structural diversity of GDB-20s and the fact that our dataset featured only a small fraction of the possibilities (Fig. 2l).

### Relevance of GDB-20s for drug discovery

Despite populating a distinct chemical space compared to known molecules, GDB-20s molecules showed favorable properties in the perspective of drug discovery. For instance, GDB-20s molecules had comparable polarity descriptor profiles (Fig. 2j and S3) and quantitative estimate of drug-likeness (QED, Fig. 2k)^64^ scores as the molecules up to the same size in the reference databases (ChEMBL ≤ 20, COCONUT ≤ 20 and ZINC ≤ 20). Along these lines, the percentage of molecules passing various drug-property filters such as Lipinski's rule of 5,^65^ Vieth,^66^ lead-like^67^ and fragment-like^68^ criteria was high and quite comparable to the percentages in the reference databases (Fig. S6a). Similarly, the frequency per molecule of the 50 most frequent functional groups found in bioactive molecules was similar in all four reference databases (Fig. S6b).^69^ Furthermore, analysis of a random sample of GDB-20s molecules showed that 98.11% passed the PAINS filters,^70^ 80.65% obeyed the Inpharmatica rule set against reactive or toxic groups,^71^ and 87.38% passed a filter reported by Freie Universität Berlin against reactive, unstable and undesirable substructures.^72^

Analysis of the 72 million Bemis–Murcko scaffolds^73^ derived from the 12 billion GDB-20s molecules revealed further aspects relevant for drug discovery. These scaffolds largely outnumbered scaffolds up to 20 atoms extracted from the reference databases ChEMBL, COCONUT and ZINC, and showed a higher Shannon entropy^74^ of scaffolds as well as higher iSIM and iSIM-σ values, indicating a higher degree of diversity (Table 4, lower part). All four scaffold datasets showed a similar power-law distribution of molecules per scaffold, with a much higher number of molecules per scaffold in GDB-20s reflecting database size (Fig. 3a). The GDB-20s data fit indicated an exponent *β* ∼0.85, extrapolating to approximately 60 billion scaffolds for the entire 32 T GDB-20 database.^75,76^

**Fig. 3:**
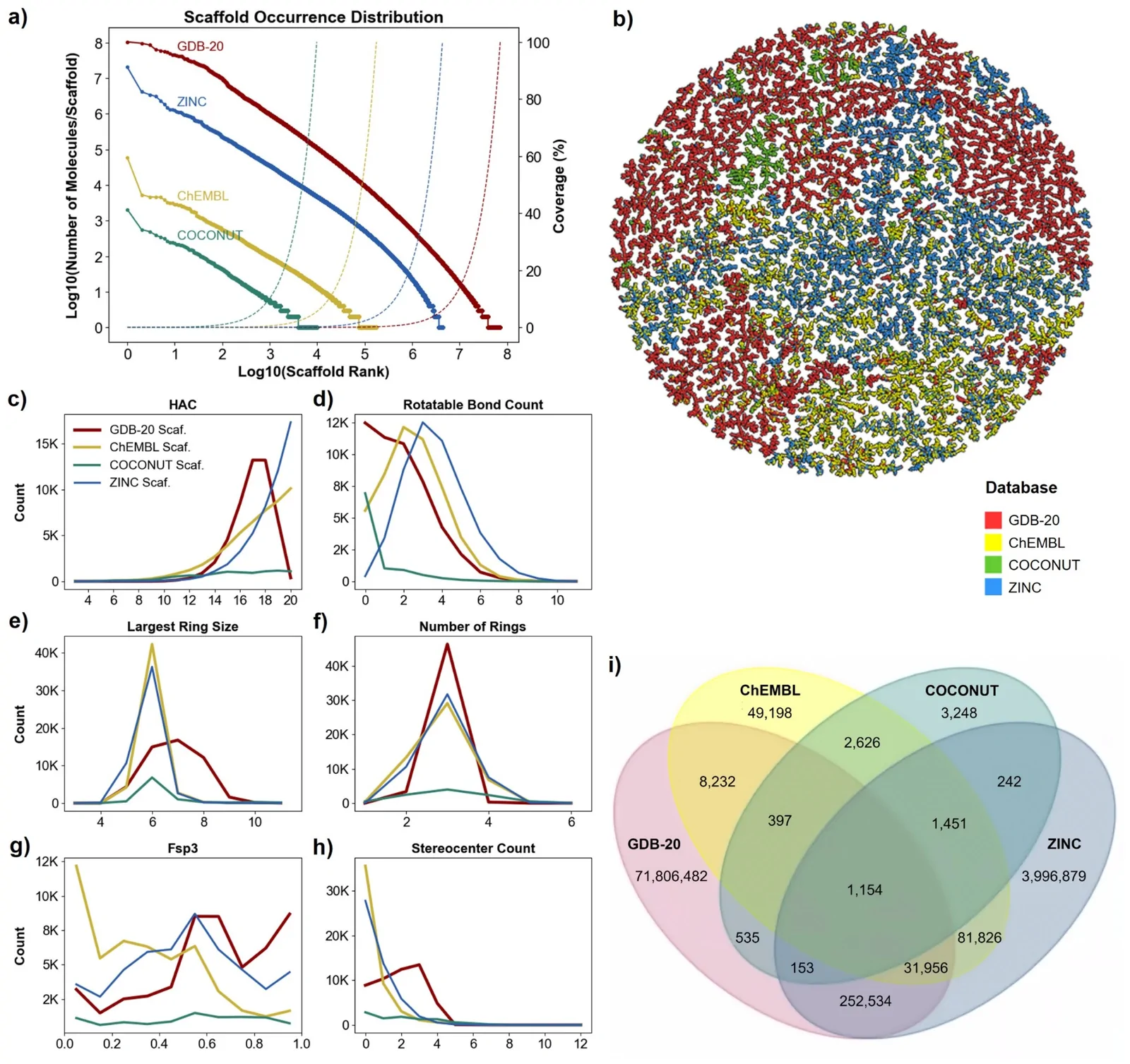
Scaffold diversity analysis of GDB-20s compared with reference compound collections, considering only scaffolds up to a heavy atom count (HAC) 20 for all reference databases: ChEMBL, COCONUT, and ZINC. (a) Scaffold occurrence distributions showing the number of molecules per Bemis–Murcko scaffold plotted against scaffold rank. The cumulative coverage of molecules by scaffolds (%) is shown on the right axis. (b) TMAP visualization of molecular scaffolds with HAC ≤ 20 sampled from GDB-20s (19 933), ChEMBL (12 431), COCONUT (2 894) and ZINC (14 296). An interactive version of the TMAPs with additional color-codes is accessible at https://tm.gdb.tools/MAP4C/GDB-20_scaffolds_across_4databases. (c–h) Distribution of key scaffold-level properties in GDB-20s compared with reference compound collections, considering only scaffolds with HAC ≤ 20. Each panel shows the property distributions for 50 000 randomly selected scaffolds from each dataset (COCONUT ≤ 20: all 9806 eligible scaffolds). Counts are shown as absolute values. Displayed properties include HAC, rotatable bond count, largest ring size, number of rings, fraction of sp^3^ carbons (*F*_sp^3^_), and stereocenter count. (i) Venn diagram of scaffold occurrence in the four databases.

Similarly to the molecules, GDB-20s scaffolds covered a distinct chemical space compared to known scaffolds (Fig. 3b). They were smaller and more rigid as estimated by the number of rotatable bonds but contained larger rings than those in reference databases, although all were all mostly tricyclic (Fig. 3c–f). Furthermore, as for molecules, the GDB-20s scaffold dataset had higher *F*_sp^3^_ and stereocenter count distribution than the reference scaffold datasets (Fig. 3g–h). Along these lines, the most frequent scaffold in GDB-20s was the 3D-shaped bicyclo[3.2.1]octane, while benzene was the most frequent scaffold in all three reference databases (Fig. S7).

Nevertheless, GDB-20s featured many scaffolds found in bioactive molecules and natural products. For instance, a Venn diagram analysis of the four scaffold collections showed a significant overlap between GDB-20s scaffolds and the reference datasets (Fig. 3i). Most notably, 41 739 scaffolds occurring in bioactive molecules from ChEMBL, representing 24.1% of ChEMBL scaffolds up to 20 atoms, occurred among the 72 million scaffolds derived from GDB-20s, even though these represented only a small fraction of the 60 billion scaffolds expected for the complete GDB-20. Note that 1 333 486 (31.6%) of the 4 222 066 scaffolds in the reference datasets were expected to occur in the full GDB-20, while the remaining 2 889 580 (68.4%) had features excluded from GDB-20 (3- or 4-membered rings, more than three rings, or various functional groups or elements excluded from GDB-20).

## Conclusion

In summary, we combined systematic graph enumeration with sampling using a graph-to-molecule transformer trained with previous GDB databases to obtain a first sample of GDB-20, which we enriched by additional sampling using recurrent neural networks. The combined dataset GDB-20s comprised 12 billion molecules, corresponding in size to 0.04% of the estimated 32 trillion molecules in the full GDB-20. By using selective graph and functional group criteria, we obtained molecules populating a chemical space of high *F*_sp^3^_, stereochemically rich molecules featuring good synthetic accessibility scores. Despite its relatively small size, our 12 billion GDB-20s sample is essentially entirely novel relative to the reference collections analyzed in this study and highly diverse in terms of molecules and scaffolds, while remaining within a property range suggesting potential utility for drug discovery. We note that GDB-20s is a model- and decoding-dependent sample rather than an unbiased or exhaustive representation of the full GDB-20 chemical space, that cross-database diversity comparisons are descriptive rather than proof of broader coverage, and that synthetic accessibility and drug-discovery relevance are inferred from computational indicators rather than validated performance. In addition, the estimated 32 trillion molecules and approximately 60 billion scaffolds for the full GDB-20 space are extrapolated values with unquantified uncertainty rather than enumerated totals. The dataset and models to generate additional GDB-20 molecules are available for virtual screening and for inspiring the synthesis of new molecules.

## Methods

### Data collection

For benchmarking and comparative analyses, we relied on the most recent public releases of several widely used chemical reference databases. Specifically, we used ChEMBL version 34 (December 2024, https://ftp.ebi.ac.uk/pub/databases/chembl/ChEMBLdb/latest) and ZINC-20 (December 2024, https://zinc.docking.org). COCONUT (September 2024, https://coconut.naturalproducts.net/download) was retrieved *via* a PostgreSQL dump. A SQL query was executed on the database to extract NPs with at least one associated citation DOI. In addition, the November 2024 release of DrugBank (https://go.drugbank.com/releases/latest) was used, comprising 1030 approved drugs with heavy atom counts up to 20. The comparison datasets were treated using a standard cheminformatics preprocessing for analyses of molecular properties, fingerprints, and scaffolds. Molecules were represented as canonical SMILES, and records containing salts, mixtures, inorganic species, or formal charges were excluded. Duplicate structures were removed using canonical SMILES as the deduplication key.

### Transformer model

The GDB20 molecular decoration transformer was developed based on the molecular transformer framework by Schwaller^77^ and the enzymatic transformer approach introduced by Kreutter.^78^ Model preprocessing, training, and validation were carried out using version 1.1.1 of OpenNMT,^79,80^ an open-source toolkit freely available on GitHub.^81^ Minor modifications were applied to the original model architectures and training procedures to tailor them to the molecular decoration task. An atom-wise, regular expression-based tokenizer^52^ was employed for input SMILES parsing, including support for multi-character tokens like Cl, Br, and bracketed atoms like [*n*H^+^]. All SMILES strings in this work were canonicalized using RDKit.^55^

Dataset splitting and generalization: to evaluate the model’s ability to generalize to unseen chemical architectures, we adopted a graph-level data split rather than a standard molecule-level split. By grouping molecules by their underlying graph types and ranking them by frequency prior to partitioning, we ensure that no graph category is shared between the training and validation sets. This protocol effectively prevents data leakage and ensures rigorous validation. The dataset was partitioned into 80% training (409 044 graphs) and 20% validation (102 260 graphs). All 26.9 million GDB-20 graphs were used as an unlabeled generation set to assess valid and unique generation across the broader chemical space, rather than translation accuracy. No separate independent final evaluation set was used.

The following hyperparameters were used for the GDB20 molecular decoration transformer model:

(1) Python preprocess.py

-train_src $DATA/src_train.txt -train_tgt $ DATA/tgt_train.txt

-valid_src $DATA/src_val.txt -valid_tgt $ DATA/tgt_val.txt

-save_data $DATA/Preprocessed

-src_seq_length 3000 -tgt_seq_length 3000

-src_vocab_size 3000 -tgt_vocab_size 3000

-share_vocab -lower

(2) Python train.py

-data $DATA/Preprocessed -save_model molecular_decoration_model

-seed 42 -train_steps 500 000 -param_init 0 -param_init_glorot

-max_generator_batches 32 -batch_size 6144

-batch_type tokens -normalization tokens -max_grad_norm 0

-accum_count 4 -optim adam -adam_beta1 0.9 -adam_beta2 0.998

-decay_method noam -warmup_steps 8000 -learning_rate 2

-label_smoothing 0.0 -layers 4 -rnn_size 384 -word_vec_size 384

-encoder_type transformer -decoder_type transformer -dropout 0.1

-position_encoding -global_attention general -global_attention_function softmax

-self_attn_type scaled-dot -heads 8 -transformer_ff 2048

-valid_steps 500 -valid_batch_size 4 -early_stopping 10

(3) Python translate. py

-model $MODEL_PATH

-batch_size 64 -replace_unk -max_length 1000

-log_probs -beam_size 300 -n_best 300

Different beam sizes were evaluated during model development, and a beam size of 300 was selected as the best practical trade-off between generation performance and computational cost. The generation of molecules was optimized for high-throughput performance, which achieved an average generation rate of 4000 molecules per second (mol s^−1^) per GPU (NVIDIA H100), 2000 mol s^−1^ (RTX 4090), or 1200 mol s^−1^ (RTX 3090). Normalized to CPU compute, this corresponds to an efficiency of 3.33 mol per s per physical core.

### RNN generative models

The model was trained based on the method introduced by Arús-Pous *et al.* in their 2019 study on randomized SMILES,^48,49^ which showed that such representations enhance the performance of molecular generative models. Together with the publication, the authors provided an open-source implementation, which was used as the foundation for training the model in this work: https://github.com/undeadpixel/reinvent-randomized.

The model was implemented in Python 3.10, following the same core architecture and training protocol. Development was carried out using the updated deep learning framework PyTorch 2.7.0.^49^ For cheminformatics tasks, the RDKit 2024.09.5 toolkit was employed,^55^ while large-scale data processing was handled with Apache Spark 3.5.5.^82^ The models were trained on NVIDIA GeForce RTX 4090 GPUs with 24 GB of GDDR6X memory.

Training was performed using the teacher forcing approach,^83^ where the correct token is provided as input at each step regardless of the model's prediction, thereby stabilizing sequence learning. Optimization was carried out with stochastic gradient descent using the Adam optimizer^84^ with parameters *β*_1_ = 0.9, *β*_2_ = 0.999, and *ε* = 10^−8^, with a batch size of 128. Model weights were updated through backpropagation, and to prevent instability during training, the computed gradients were normalized such that their norm was constrained to 1.0.

### Sampling stratification

A stratified sampling strategy based on Heavy Atom Count (HAC) ratios was employed to construct a representative subset of GDB-20 for downstream characterization. This approach preserved structural diversity while accurately capturing the database's overall generative trends. In total, 50 000 molecules were drawn from GDB-20s for comparison against reference collections. To maintain consistency in analysis scale, external datasets exceeding 50 000 molecules were randomly downsampled to this size, whereas smaller datasets were analyzed in their entirety. Random subsampling for the molecular-property, molecular-shape, medicinal-chemistry-rule, functional-group and scaffold-property analyses was performed using a fixed random seed of 42.

### Stereochemical handling

The analyses were conducted primarily at the level of molecular constitution. Stereoisomers were not systematically enumerated or treated as separate GDB-20s records. Stereochemical richness was quantified by counting both assigned and unassigned potential stereogenic centres using RDKit with include unassigned = true. Where stereochemical annotations were present in the input SMILES, they were retained during RDKit parsing and used by stereochemistry-sensitive calculations.

### TMAPs

Tree-maps (TMAPs)^58^ were generated by specifying standard parameters, using the MAP4C (MinHashed atom-pair fingerprint up to a diameter of four bonds, including chirality) molecular fingerprint.^59^ MAP4C fingerprints were computed with a dimension of 256 and indexed using an LSH forest with 128 prefix trees. The layout parameters were set to *k* = 20, *k*_c_ = 50, node_size = 1/26, sl_repeats = 2, sl_extra_scaling_steps = 5, and mmm_repeats = 2. The interactive Faerun visualization used a point scale of 3.2 and spherical point shading.

### 3D conformer generation

For the molecular-shape analysis in Fig. S4, stereochemical annotations present in the input SMILES were retained, without enumerating unspecified stereoisomers. Explicit hydrogens were added, and one conformer per molecule was generated using RDKit ETKDG version 1 and subjected to UFF optimization using default settings (maximum 200 iterations; van der Waals threshold 10.0). NPR1 and NPR2 were calculated as *I*_1_/*I*_3_ and *I*_2_/*I*_3_, respectively. No explicit seed was set for conformer generation; the RDKit default randomSeed = −1 was used.

## Author contributions

YB: conceptualization, data curation, formal analysis, investigation, methodology, software, validation, visualization, writing – original draft, writing – review & editing. SJ: methodology, software, validation, writing – review & editing. JLR: conceptualization, funding acquisition, project administration, resources, supervision, writing – original draft, writing – review & editing. All authors read and approved the final manuscript.

## Conflicts of interest

There are no conflicts to declare.

## Supplementary Material

DD-OLF-D6DD00213G-s001

## Data Availability

The GDB-20s database is hosted on the open-access repository Zenodo, with all molecules provided in canonical SMILES format and compressed as a GNU zip archive. To manage the deduplication of 12 billion entries, we utilized a canonicalization and parallelized hashing pipeline, resulting in highly compressed data that remains within manageable storage limits. The complete GDB-20s dataset is provided as four compressed files (GDB20.HAC1–17.tar.gz, 2.8 GB; GDB20.HAC18.tar.gz, 13.7 GB; GDB20.HAC19.tar.gz, 17.9 GB; and GDB20.HAC20.tar.gz, 35.3 GB), corresponding to molecules grouped by heavy atom count (HAC). Within the archives, the file names describe the origin and processing status of each subset: for example, *nodes1_17* denotes molecules generated from graphs with 1–17 nodes; *unique_canonical_SMILES_only* indicates that the file contains only unique canonical SMILES, one per line, without additional columns or molecular identifiers; *sorted_dedup* indicates that the file was sorted and deduplicated; *part_ab* identifies one split part of a large file; and *hac1-17* denotes the corresponding heavy-atom-count range. This HAC-indexed partitioning allows users to access specific molecular size regimes without downloading the entire database, ensuring the resource is accessible for both local desktop analysis and large-scale server-side processing. Representative samples of 50 000 (470 KB) and 50 000 000 molecules (484 MB), are also included. All files are available for download at https://doi.org/10.5281/zenodo.17368725 (part1), https://doi.org/10.5281/zenodo.17375415 (part 2), and can be accessed *via* the Reymond Group website (https://gdb.unibe.ch/downloads/). Use of GDB-20s, in whole or in part, in connection with patent applications is prohibited, and the database or large portions thereof must not be redistributed without the express written permission of the authors. All relevant code developed in this work has been released under the MIT license. The exact code version associated with this study is archived at Zenodo (https://doi.org/10.5281/zenodo.23026437, release v1.0.1) and corresponds to tag v1.0.1 of the GitHub repository (https://github.com/reymond-group/GDB-ML). The archived v1.0.1 code release contains the tokenized transformer source-graph and target-molecule data as split files under transformer/gdb20_data/. After concatenation, these files form the actual OpenNMT inputs: src_train.txt, tgt_train.txt, src_val.txt, and tgt_val.txt. The release also contains the one-million-molecule training and validation SMILES sets for the five RNN models under generative_models/gdb20_data/, covering HAC 1–17, HAC 18, HAC 19, and two HAC 20 subsets. The trained transformer and RNN model files are provided under transformer/gdb20_model/ and generative_models/gdb20_models/, respectively. The current archive does not cover regeneration of the earlier graph-selection stage, which is described in the manuscript and documented in the repository's REPRODUCIBILITY.rst file. Supplementary information (SI): molecule-per-graph extrapolation, SMILES-length distributions, model evaluations, and additional analyses of molecular properties, shape, approved-drug isomers, medicinal-chemistry rules, functional groups, and scaffolds. See DOI: https://doi.org/10.1039/d6dd00213g.
