# Supplementary material for: Sampling a trillion-sized GDB-20 database of drug-like molecules by generative artificial intelligence
Source: Digit Discov. 2026 Oct 16. Online ahead of print. doi: 10.1039/d6dd00213g (PMC13711157; doi:10.1039/d6dd00213g)
Supplement: Supplementary file 1 [file DD-OLF-D6DD00213G-s001.pdf]

Supplementary Information (SI) for Digital Discovery.
This journal is © The Royal Society of Chemistry

Supporting Information for

Sampling a Trillion-Sized GDB-20 Database of Drug-Like Molecules by Generative Artificial Intelligence

Ye Buehler,[a] Sacha Javor,[a] and Jean-Louis Reymond*[a]

[a)] *Department of Chemistry, Biochemistry and Pharmaceutical Sciences, University of Bern, Freiestrasse 3, 3012 Bern, Switzerland*

e-mail: jean-louis.reymond@unibe.ch

Contents

Figure S1 2

Figure S2 2

Table S1 3

Figure S3 4

Figure S4 4

Figure S5 5

Table S2 6

Figure S6 7

Figure S7 8

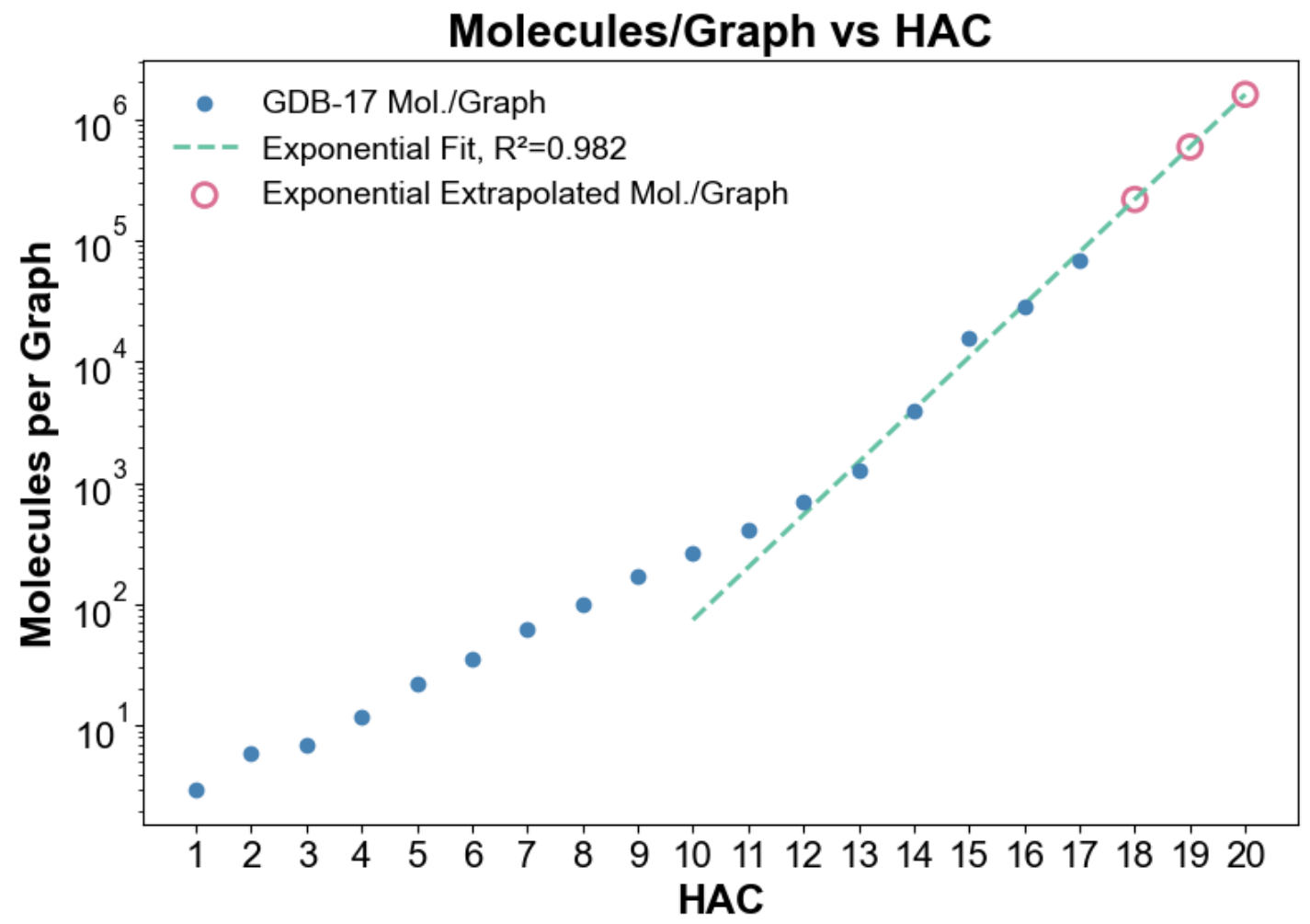

Figure S1. Number of molecules per graph as a function of heavy atom count (HAC) in GDB-17. Data taken from Ref. [19] The number of molecules per graph increases faster above HAC=13 due to more restrictive graph selection rules removing strained graphs with small rings, for which fewer molecules are possible due to functional group selection rules for small rings. The data above HAC13 follow an exponential trend (R^2 = 0.982). Extrapolation to HAC 18–20 (open circles) combined with the number of GDB-20 graphs provides an estimate of the total achievable GDB-20 size through generative models.

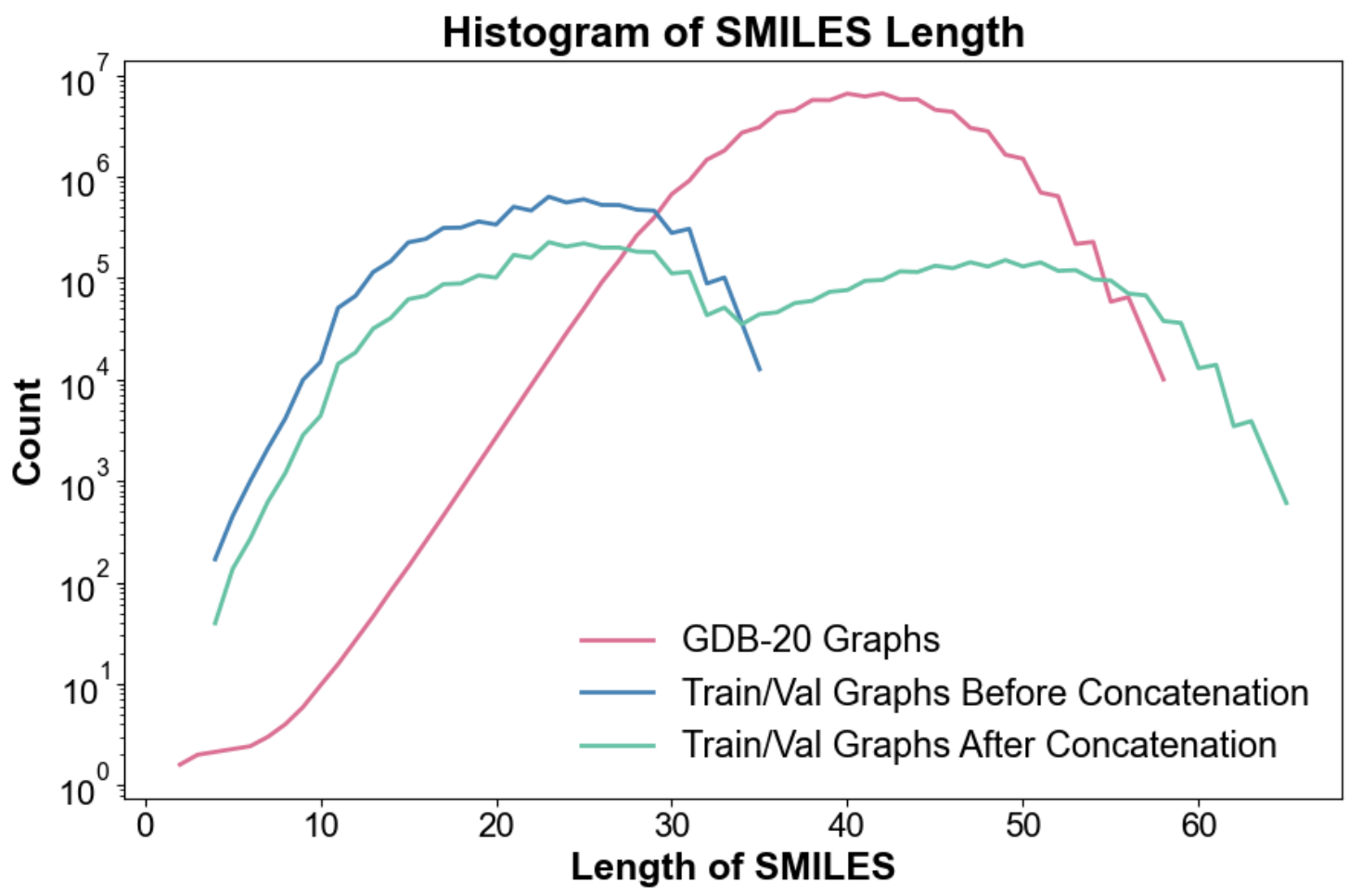

Figure S2. SMILES length histogram for saturated hydrocarbons in the training set, concatenated training set, and in the GDB-20 hydrocarbons.

Table S1. Intrinsic evaluation of generated SMILES across graph node subsets for all models.

			Transformer model		
**Nodes**[a]	**1-17**	**18**	**19**	**20**	**Overall**[i]
**Novel**[b]	212,471,570	374,214,271	1,413,617,970	3,291,626,531	5,291,930,342
**% Novelty**	99.59%	≈100%	≈100%	≈100%	99.98%
**Mean NLL**[c]	7.43	8.01	8.48	8.96	8.22
**NLL SD**[d]	1.10	1.06	1.06	1.08	1.21
**Histo. Entropy**[e]	2.32	2.33	2.34	2.19	2.27
**KL Divergence**[f]	5.69	5.84	5.84	4.82	4.46
**Unif**[g]	0.41	0.41	0.41	0.39	0.40
**LL Entropy**[h]	19.32	19.33	19.27	19.22	21.10
			RNN generative models		
**Nodes**[a]	**1-17**	**18**	**19**	**20**	**Overall**[i]
**Novel**[b]	624,039,818	1,350,158,537	2,548,343,962	3,620,807,816	8,143,350,133
**% Novelty**	99.79%	99.91%	99.97%	99.98%	99.95%
**Mean NLL**[c]	26.22	27.38	29.12	29.84	28.14
**NLL SD**[d]	1.59	1.67	1.80	2.20	2.32
**Histo. Entropy**[e]	2.46	2.60	2.48	2.70	2.55
**KL Divergence**[f]	3.91	4.16	3.22	2.93	2.32
**Unif**[g]	0.44	0.46	0.44	0.48	0.45
**LL Entropy**[h]	18.78	18.71	16.68	18.08	19.91

[a]Number of graph nodes considered. [b]Number of unique molecules not appearing in either the training or validation sets. [c]Average negative log-likelihood (NLL) of generated molecules. [d]Standard deviation of NLL across generated SMILES. [e]Histogram entropy, refers to the Shannon entropy of the empirical SMILES-token frequency distribution. [f]Kullback–Leibler divergence between the generated SMILES-token distribution and the training distribution. [g]Uniformity score quantifying how evenly probability mass is spread across generated molecules. [h]Entropy of the log-likelihood distribution across generated molecules. [i]Counts in the overall column represent sums of the absolute counts, whereas percentages are reported as weighted averages across all node subsets. Statistical metrics in the overall column were calculated from pooled observations across all node subsets. Overall, the transformer model exhibits stable distributional behavior across node subsets, with consistent histo. entropy and LL entropy, decreasing KL divergence, and nearly constant Unif, indicating good alignment with the training distribution while maintaining a balanced and diverse generation profile. In contrast, RNN generative models exhibit substantially higher mean NLL and NLL SD, together with greater variability in histo. entropy and LL entropy across node subsets.

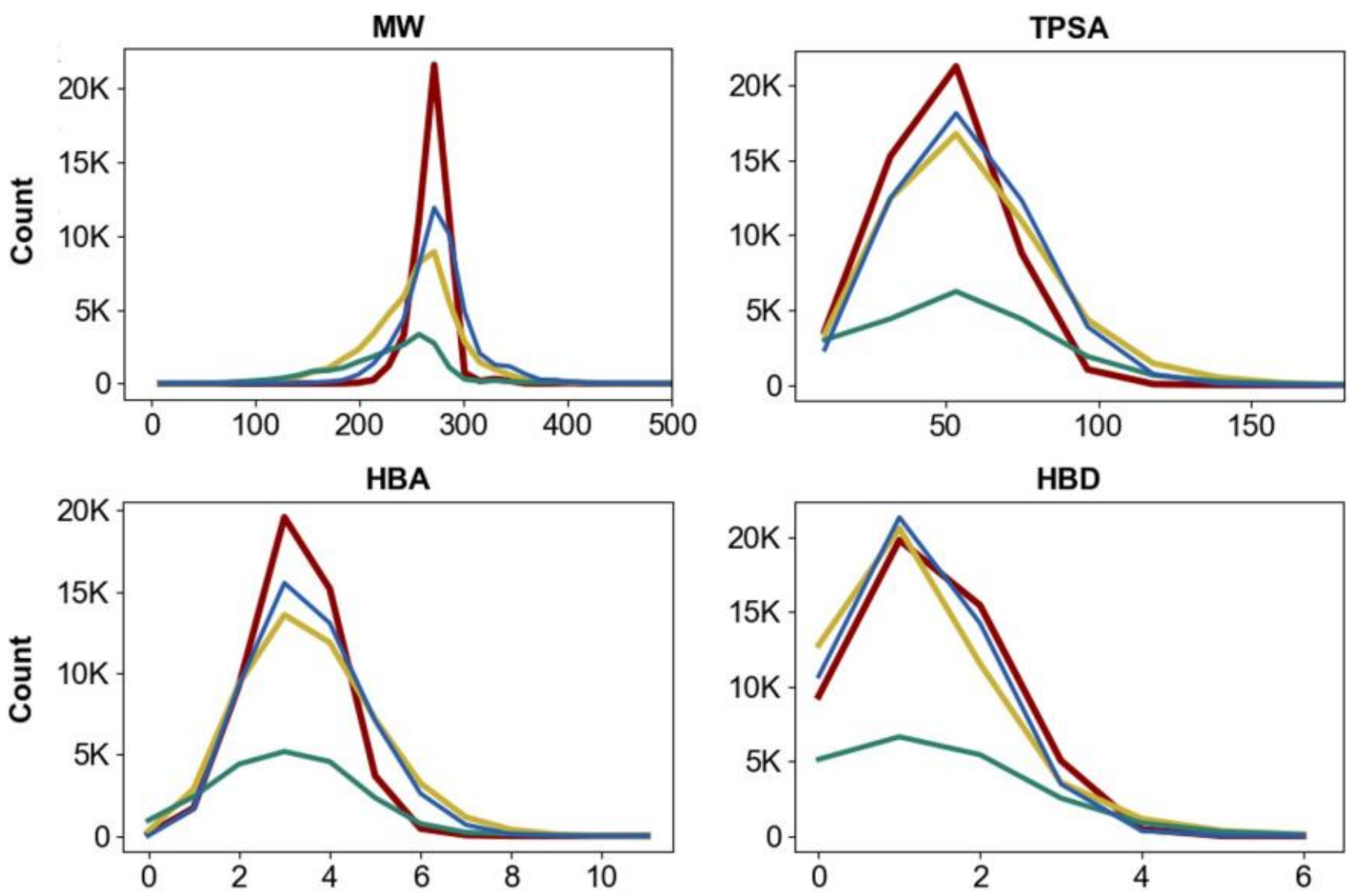

Figure S3. Distribution of key molecular properties in GDB-20s compared with reference compound collections capped at HAC≤20. Each panel shows the property distributions for GDB-20s (red), ChEMBL≤20 (yellow), COCONUT≤20 (green), and ZINC≤20 (blue), with 50,000 randomly selected compounds analyzed from each dataset. Properties include molecular weight (MW), topological polar surface area (TPSA), hydrogen bond acceptors (HBA), and hydrogen bond donors (HBD).

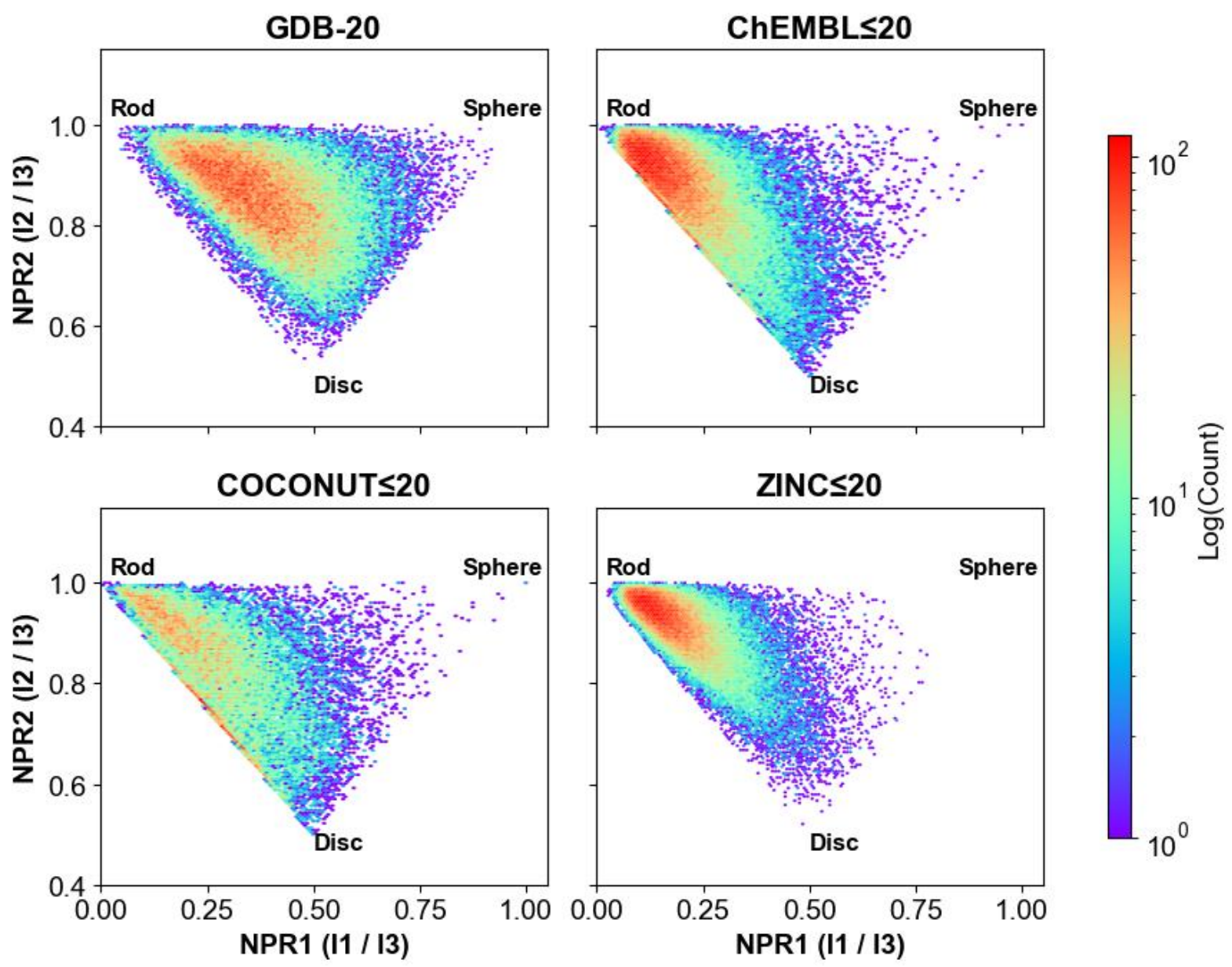

Figure S4. Molecular shape analysis based on principal moments of inertia (PMI), shown as a shape occupancy map across rod-, disc-, and sphere-like geometries.

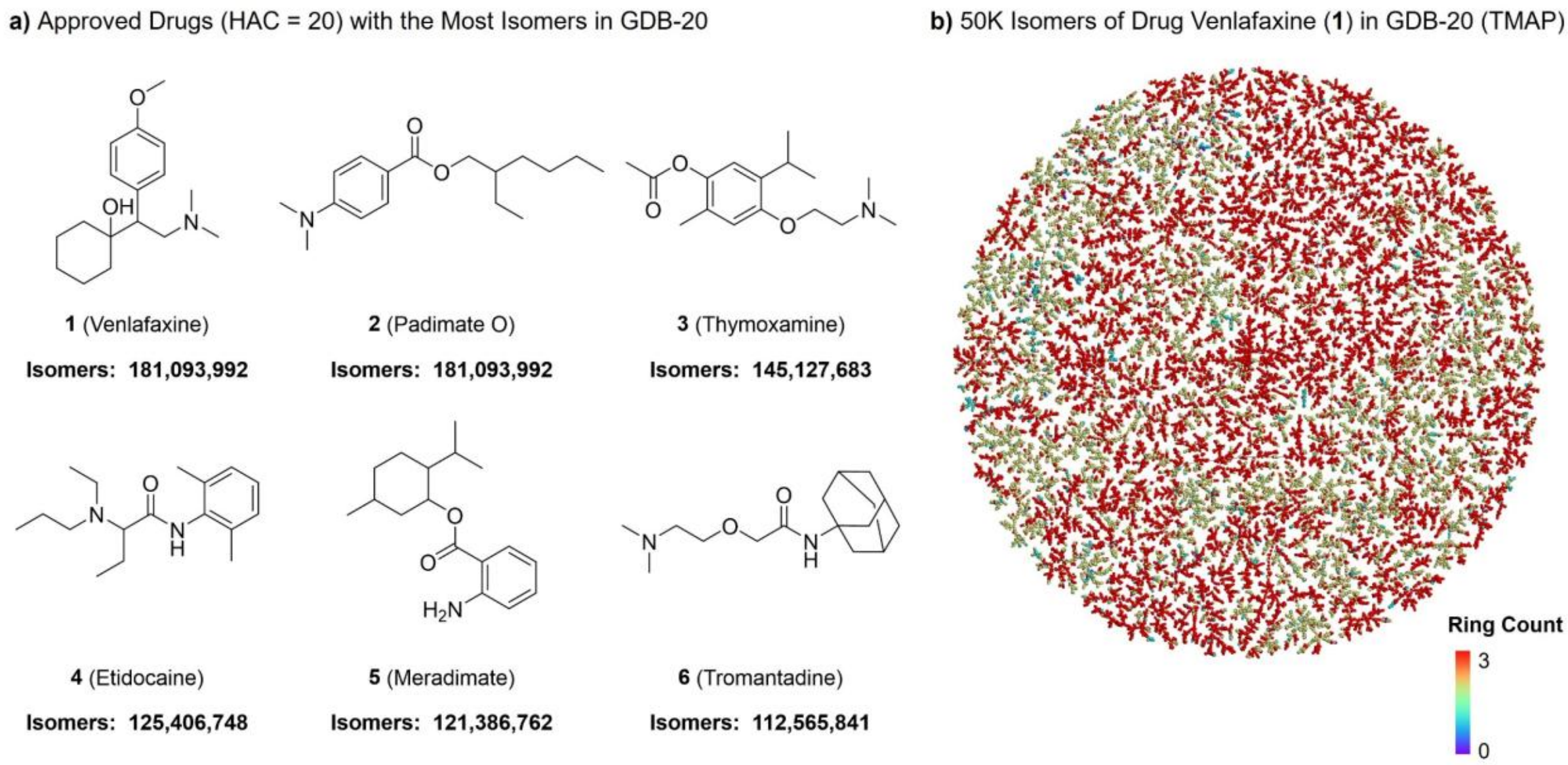

Figure S5. (**a**) Approved drugs (HAC = 20) from DrugBank with the highest number of constitutional isomers in GDB-20. The total number of corresponding GDB-20 isomers for each compound is indicated below the respective structures. (**b**) TMAP visualization of 50,000 random isomers of the approved DrugBank compound Venlafaxine (**1**), which has the highest number of constitutional isomers in GDB-20, when HAC = 20. Each point represents one isomer, colored according to its ring count (0–3). An interactive version of the TMAPs with additional color-codes is accessible at https://tm.gdb.tools/MAP4C/GDB-20_50K_isomers_of_Venlafaxine.

Table S2. Constitutional isomers of approved drugs found in GDB-20 (HAC=20).

No.	Drug Names[a]	Molecular formula	DrugBank ID	Number of isomers
1	Venlafaxine	$C_{17}H_{27}NO_2$	DB00285	181'093'992
2	Padimate O	$C_{17}H_{27}NO_2$	DB11570	181'093'992
3	Thymoxamine	$C_{16}H_{25}NO_3$	DB09205	145'127'683
4	Etidocaine	$C_{17}H_{28}N_2O$	DB08987	125'406'748
5	Meradimate	$C_{17}H_{25}NO_2$	DB11096	121'386'762
6	Tromantadine	$C_{16}H_{28}N_2O_2$	DB13288	112'565'841
7	Molindone	$C_{16}H_{24}N_2O_2$	DB01618	91'029'772
8	Ropivacaine	$C_{17}H_{26}N_2O$	DB00296	73'306'146
9	Menthyl Salicylate	$C_{17}H_{24}O_3$	DB11201	72'152'923
10	Cyclandelate	$C_{17}H_{24}O_3$	DB04838	72'152'923
11	Linoleic Acid	$C_{18}H_{32}O_2$	DB14104	57'790'207
12	Homatropine	$C_{16}H_{21}NO_3$	DB11181	57'760'804
13	Linolenic Acid	$C_{18}H_{30}O_2$	DB00132	52'676'811
14	Gamolenic Acid	$C_{18}H_{30}O_2$	DB13854	52'676'811
15	Dextromethorphan	$C_{18}H_{25}NO$	DB00514	45'821'519
16	Dihydroartemisinin	$C_{15}H_{24}O_5$	DB11638	30'278'060
17	Doxylamine	$C_{17}H_{22}N_2O$	DB00366	29'813'712
18	Laurocapram	$C_{18}H_{35}NO$	DB17308	27'438'856
19	Estradiol	$C_{18}H_{24}O_2$	DB00783	24'289'857
20	Physostigmine	$C_{15}H_{21}N_3O_2$	DB00981	23'782'742
21	Mavacamten	$C_{15}H_{19}N_3O_2$	DB14921	19'210'876
22	Fenpropidin	$C_{19}H_{31}N$	DB12728	17'146'806
23	Oleic Acid	$C_{18}H_{34}O_2$	DB04224	16'007'724
24	Orphenadrine	$C_{18}H_{23}NO$	DB01173	15'398'045
25	Perhexiline	$C_{19}H_{35}N$	DB01074	14'122'360
26	Pipradrol	$C_{18}H_{21}NO$	DB11584	8'907'819
27	Estrone	$C_{18}H_{22}O_2$	DB00655	7'868'428
28	Carbinoxamine	$C_{16}H_{19}ClN_2O$	DB00748	6'623'098
29	Cyclizine	$C_{18}H_{22}N_2$	DB01176	5'821'927
30	Desipramine	$C_{18}H_{22}N_2$	DB01151	5'821'927
31	N-Cyclohexyl-N'-phenyl-1,4-phenylenediamine	$C_{18}H_{22}N_2$	DB14196	5'821'927
32	Oxamniquine	$C_{14}H_{21}N_3O_3$	DB01096	5'723'399
33	Clofedanol	$C_{17}H_{20}ClNO$	DB04837	4'577'306
34	Apomorphine	$C_{17}H_{17}NO_2$	DB00714	3'469'959
35	Diethylstilbestrol	$C_{18}H_{20}O_2$	DB00255	3'299'119
36	Mirtazapine	$C_{17}H_{19}N_3$	DB00370	3'047'334
37	Antazoline	$C_{17}H_{19}N_3$	DB08799	3'047'334
38	Promethazine	$C_{17}H_{20}N_2S$	DB01069	2'506'841
39	Promazine	$C_{17}H_{20}N_2S$	DB00420	2'506'841
40	Nevirapine	$C_{15}H_{14}N_4O$	DB00238	2'444'312
41	Mianserin	$C_{18}H_{20}N_2$	DB06148	1'982'036
42	Pitolisant	$C_{17}H_{26}ClNO$	DB11642	1'713'499
43	Rizatriptan	$C_{15}H_{19}N_5$	DB00953	1'669'315
44	Diamthazole	$C_{15}H_{23}N_3OS$	DB13858	1'582'079
45	Sumatriptan	$C_{14}H_{21}N_3O_2S$	DB00669	1'376'908
46	Stearic Acid	$C_{18}H_{36}O_2$	DB03193	1'093'625
47	Asenapine	$C_{17}H_{16}ClNO$	DB06216	1'064'233
48	Protriptyline	$C_{19}H_{21}N$	DB00344	780'599
49	Nortriptyline	$C_{19}H_{21}N$	DB00540	780'599
50	Bromazine	$C_{17}H_{20}BrNO$	DB01237	771'662
51	Dienestrol	$C_{18}H_{18}O_2$	DB00890	729'888
52	Metoclopramide	$C_{14}H_{22}ClN_3O_2$	DB01233	682'580
53	Diazepam	$C_{16}H_{13}ClN_2O$	DB00829	666'601
54	Mazindol	$C_{16}H_{13}ClN_2O$	DB00579	666'601
55	Thenalidine	$C_{17}H_{22}N_2S$	DB04826	643'538
56	Pentoxifylline	$C_{13}H_{18}N_4O_3$	DB00806	599'643
57	Lomifylline	$C_{13}H_{18}N_4O_3$	DB18002	599'643

No.	Drug Names[a]	Molecular formula	DrugBank ID	Number of isomers
58	Oxazepam	$C_{15}H_{11}ClN_2O_2$	DB00842	407'162
59	Phenindamine	$C_{19}H_{19}N$	DB01619	221'319
60	Pomalidomide	$C_{13}H_{11}N_3O_4$	DB08910	202'083
61	Tolcapone	$C_{14}H_{11}NO_5$	DB00323	76'075
62	Sulfacytine	$C_{12}H_{14}N_4O_3S$	DB01298	59'277
63	Entecavir	$C_{12}H_{15}N_5O_3$	DB00442	56'420
64	Imipenem	$C_{12}H_{17}N_3O_4S$	DB01598	44'410
65	Bromfenac	$C_{15}H_{12}BrNO_3$	DB00963	42'788
66	Sertraline	$C_{17}H_{17}Cl_2N$	DB01104	32'964
67	Flortaucipir	$C_{16}H_{10}FN_3$	DB15033	1'127
68	Tafamidis	$C_{14}H_7Cl_2NO_3$	DB11644	909
69	Lumiracoxib	$C_{15}H_{13}ClFNO_2$	DB01283	671
70	Flufenamic Acid	$C_{14}H_{10}F_3NO_2$	DB02266	315
71	Tazobactam	$C_{10}H_{12}N_4O_5S$	DB01606	178
72	Trifluridine	$C_{10}H_{11}F_3N_2O_5$	DB00432	32
73	Chloramphenicol	$C_{11}H_{12}Cl_2N_2O_5$	DB00446	29

[a] Common approved drugs found in the DrugBank database. DrugBank IDs link to the corresponding DrugBank records.

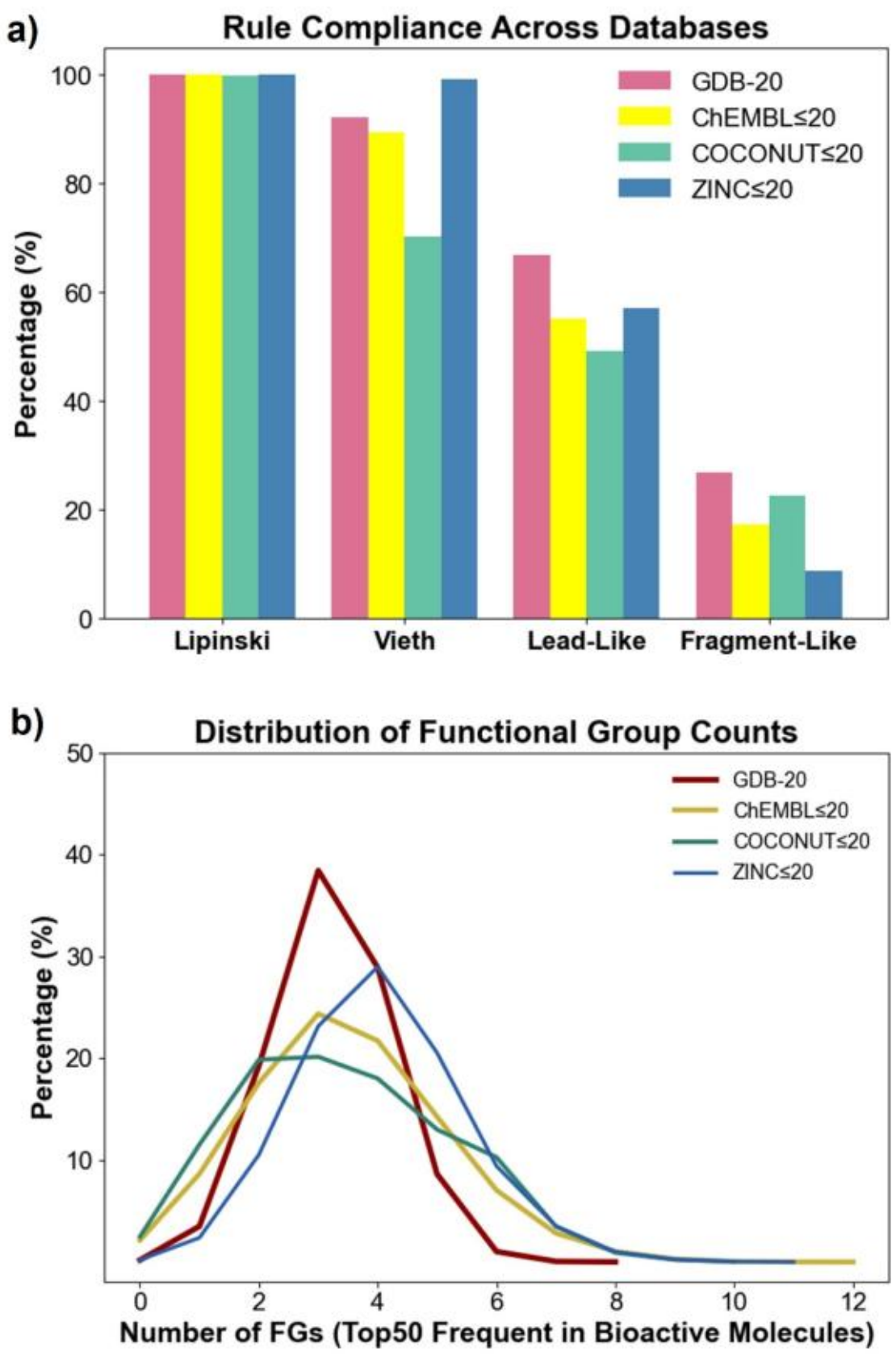

Figure S6. (**a**) Compliance with common medicinal chemistry rules across databases. The percentages of molecules in GDB-20, ChEMBL≤20, COCONUT≤20, and ZINC≤20 satisfying Lipinski's Rule of Five, Vieth's criteria, lead-like rules, and fragment-like rules are shown. (**b**) Histogram of functional group counts per molecule, based on the top 50 functional groups most frequently observed in bioactive molecules.

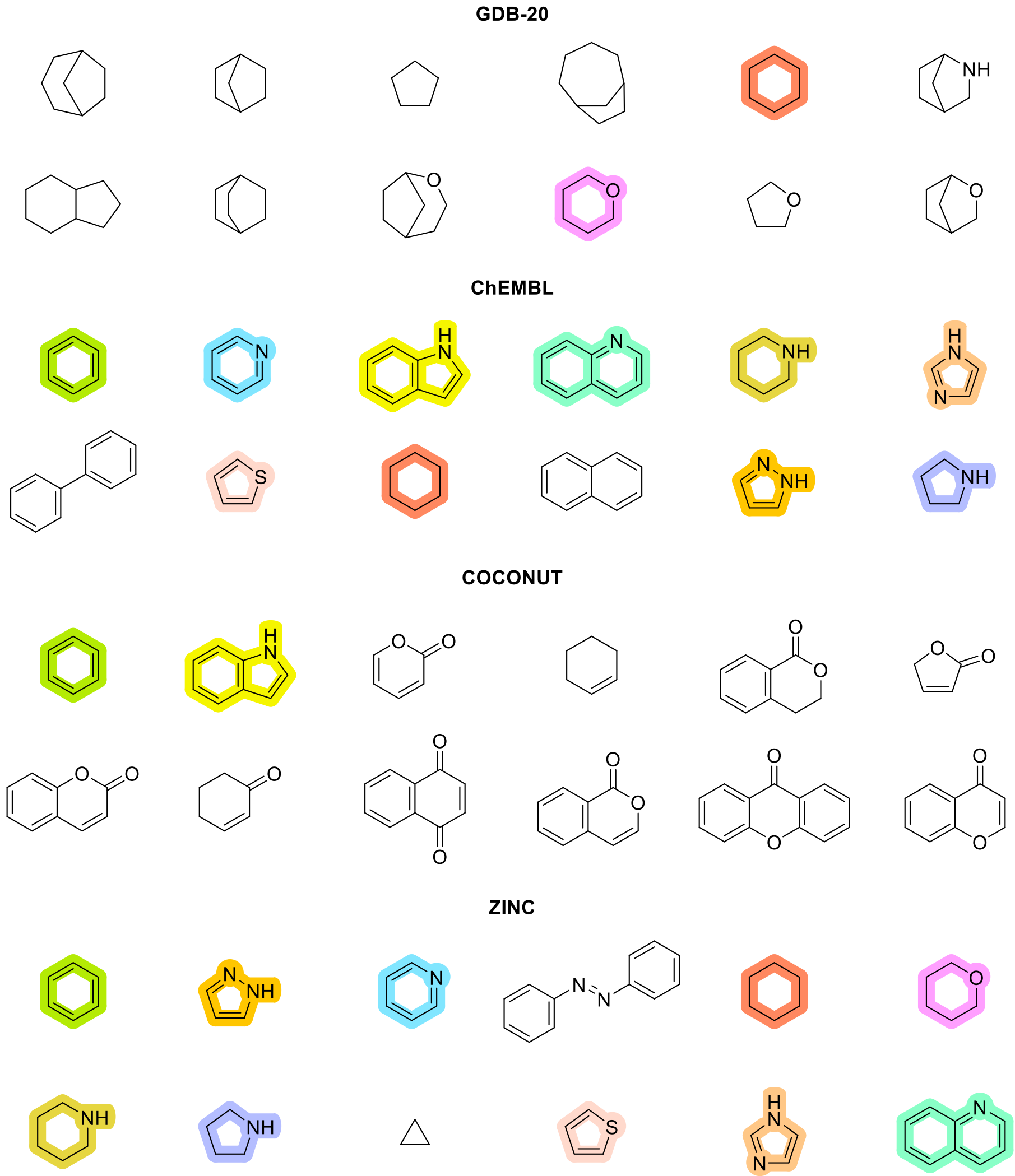

Figure S7. Top 12 most frequent Bemis–Murcko scaffolds among 50,000 sampled molecules from each dataset (GDB-20, ChEMBL, COCONUT, and ZINC). Scaffolds occurring in the top-12 list of more than one dataset are highlighted in color.
